# Fatty acid‐based diet estimates suggest ringed seal remain the main prey of southern Beaufort Sea polar bears despite recent use of onshore food resources

**DOI:** 10.1002/ece3.6043

**Published:** 2020-01-29

**Authors:** Jennifer Bourque, Todd C. Atwood, George J. Divoky, Connie Stewart, Melissa A. McKinney

**Affiliations:** ^1^ Wildlife and Fisheries Conservation Center Department of Natural Resources and the Environment and Center for Environmental Sciences and Engineering University of Connecticut Storrs CT USA; ^2^ Alaska Science Center United States Geological Survey Anchorage AK USA; ^3^ Cooper Island Arctic Research Seattle WA USA; ^4^ Department of Mathematics and Statistics University of New Brunswick Saint John NB Canada; ^5^ Department of Natural Resource Sciences McGill University Ste‐Anne‐de‐Bellevue QC Canada

**Keywords:** black guillemot, bowhead whale, feeding ecology, sea ice loss, *Ursus maritimus*

## Abstract

Polar bears (*Ursus maritimus*) from the southern Beaufort Sea (SB) subpopulation have traditionally fed predominantly upon ice‐seals; however, as the proportion of the subpopulation using onshore habitat has recently increased, foraging on land‐based resources, including remains of subsistence‐harvested bowhead whales (*Balaena mysticetus*) and colonial nesting seabirds has been observed. Adipose tissue samples were collected from this subpopulation during the springs of 2013–2016 and analyzed for fatty acid signatures. Diet estimates were generated for the proportional consumption of ringed seal (*Pusa hispida*), bearded seal (*Erignathus barbatus*), and beluga whale (*Delphinapterus leucas*), relative to onshore foods, including bowhead whale remains and seabird, as represented by black guillemot (*Cepphus grylle mandtii*) nestlings and eggs. Quantitative fatty acid signature analysis (QFASA) estimated that the ice‐obligate prey, ringed seal, remained the predominant prey species of SB polar bears (46.4 ± 1.8%), with much lower consumption of bearded seal (19.6 ± 2.0%), seabird (17.0 ± 1.2%), bowhead whale (15.0 ± 1.4%), and hardly any beluga whale (2.0 ± 0.5%). Adult and subadult females appeared to depend more on the traditional ringed seal prey than adult and subadult males. Diet estimates of SB polar bears showed significant interannual variability for all prey (*F*_12, 456_ = 3.17, *p* < .001). Longer‐term estimates suggested that both types of onshore prey, bowhead whale remains and seabird, have represented a moderate proportion of the food resources used by SB polar bears since at least the start of the 21st Century.

## Graphical abstract

Polar bears in the southern Beaufort Sea are increasingly using land‐based habitat. This work suggests that these polar bears still rely heavily on ringed seal, although onshore seabirds and whale carcasses have represented a moderate proportion of their diet since at least the turn of the 21st century.

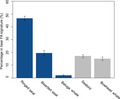

## 1 INTRODUCTION

Polar bears (*Ursus maritimus*) serve as the apex predator of Arctic marine ecosystems and as a sentinel species of ecological change in the North (Moore, 2008). These large carnivores are considered sea ice‐associated predators, but declines in the availability of sea ice habitat have resulted in increased use of onshore habitat during the reduced ice season (Atwood et al., 2016; Cherry, Derocher, Thiemann, & Lunn, 2013). This seasonal transition to terrestrial habitat use raises questions about food availability and energy intake provided by onshore resources relative to that necessary to maintain the energetically demanding lifestyle of a polar bear (Pagano et al., 2018; Rode, Robbins, Nelson, & Amstrup, 2015; Rode et al., 2018). Much research has sought to increase knowledge of the feeding habits of polar bears that increasingly use onshore habitats (Gormezano, Ellis‐Felege, Iles, Barnas, & Rockwell, 2017; Iverson, Gilchrist, Smith, Gaston, & Forbes, 2014; McKinney, Atwood, Iverson, & Peacock, 2017). Improved understanding of the recent foraging habits of these top predators may reveal broader fluctuations in the Arctic food web as a result of environmental change, namely sea ice loss (Rode et al., 2018).

The southern Beaufort Sea (SB) subpopulation of polar bears is currently estimated at 600–1,200 individuals (Bromaghin et al., 2015) and has decreased in size over the past three decades concomitant with sea ice loss (Bromaghin et al., 2015; Regehr, Hunter, Caswell, Amstrup, & Stirling, 2010). Reduced body size and recruitment in SB polar bears have also been associated with the decline of sea ice cover (Rode, Amstrup, & Regehr, 2010). Sea ice has historically been available to polar bears in the SB throughout the year, but in recent years the sea ice edge has retreated north of the continental shelf in the summer‐fall and toward deeper, less productive waters of the Arctic Ocean (Dunton, Goodall, Schonberg, Grebmeier, & Maidment, 2005). During the annual sea ice retreat, some SB polar bears have remained on the ice, while others have come ashore, with increasing proportions reported onshore since the year 2000 (Atwood et al., 2016; Schliebe et al., 2008).

The increased reliance on coastal onshore habitat as sea ice has declined may affect the feeding ecology of SB polar bears. On‐ice habitat provides polar bears with access to their preferred prey: ringed seals (*Pusa hispida*), bearded seals (*Erignathus barbatus*), and occasionally, beluga whales (*Delphinapterus leucas*; Stirling & Archibald, 1977; Thiemann, Iverson, & Stirling, 2008). SB polar bears that come ashore instead of remaining with the retreating pack ice have been documented feeding on alternative land‐based foods (McKinney et al., 2017; Rogers, Peacock, Simac, O'Dell, & Welker, 2015), a behavior that has also shown recent increases in some Canadian subpopulations (Galicia, Thiemann, Dyck, Ferguson, & Higdon, 2016; Gormezano & Rockwell, 2013; Iverson et al., 2014; Smith, Elliott, Gaston, & Gilchrist, 2010). Bowhead whales (*Balaena mysticetus*) are harvested for subsistence in the SB and in other subpopulations including Foxe Basin (FB), wherein bowhead remains washing ashore have also been attributed to predation by killer whales (*Orcinus orca*) in recent years (Galicia et al., 2016). These remains are foraged upon by polar bears in both subpopulations (Atwood et al., 2016; Galicia et al., 2016), and elsewhere where beached whales are found (Laidre, Stirling, Estes, Kochnev, & Roberts, 2018).

Predation on seabirds has also been observed with increasing frequency in a few subpopulations (e.g., western Hudson Bay [WHB], Davis Strait [DS], Foxe Basin [FB]), as polar bear onshore arrival now overlaps with seabird nesting activities to a greater degree (Dey et al., 2017, 2018; Gormezano & Rockwell, 2013; Iverson et al., 2014). There are 50 million or so seabirds that nest in more than 1,600 colonies on Alaska's coasts in the summer season, representing about 87% of total number of seabirds in the United States (U.S. Fish & Wildlife Service, 2019). In the Beaufort Sea of Alaska, the density of pelagic seabirds within 100 × 100 km grids was estimated to be >280 kg/km^2^, ranking among the highest across Alaskan marine regions (Drew, 2015) and also, for the western range of the SB subpopulation, among the highest in the North American Arctic (Gall, Day, & Morgan, 2013; Wong, Gjerdrum, Morgan, & Mallory, 2014). If seabird density is relatively consistent at a coarse scale (e.g., 100 km × 100 km grid cell), the estimated seabird biomass distributed over a >50,000 km^2^ area spanning the western (−161°W) and eastern (−133°W) boundaries of the SB subpopulation could approach 14,000,000 kg. However, all of those seabirds are not readily accessible to polar bears as studies suggest most predation occurs at nesting colonies on eggs and nestlings, and also on molting/flightless adults (Divoky, Lukacs, & Druckenmiller, 2015; Iles, Peterson, Gormezano, Koons, & Rockwell, 2013; Iverson et al., 2014). Polar bears in the SB have been observed feeding on a breeding colony of black guillemot (*Cepphus grylle mandtii*) on Cooper Island largely post‐2002, and until the nests were protected in 2010 by researchers (Divoky et al., 2015), and on common eider nests (*Somateria mollissima*; U.S. Fish and Wildlife Service, unpublished data). Despite these observations of polar bear predation on seabirds and sea ducks in multiple regions, their contribution to the diet has not been estimated for any polar bear subpopulation to date.

For polar bears, the energetic inputs from lipid‐rich, ice‐associated prey species are crucial for the high‐energy lifestyles that come along with living in the Arctic (Rode et al., 2015). Accordingly, it has been posited that onshore resources are not important from the perspective of energy intake, but few have been included in quantitative diet estimates (Dey et al., 2017; Pilfold et al., 2016; Rode et al., 2015). While bowhead whale has been recently included in both fatty acid and stable isotope‐based diet estimates (Cherry, Derocher, Hobson, Stirling, & Thiemann, 2011; McKinney et al., 2017; Rogers et al., 2015), seabird has not yet been considered. Here, our objective is to use fatty acid signatures to quantitatively estimate the extent of SB polar bear feeding on onshore prey including seabird and bowhead whale from 2013 to 2016, relative to traditional prey of ringed seal, bearded seal, and beluga whale. We also combine our fatty acid data with previous work from 2004 to 2012 (McKinney et al., 2017) to assess temporal trends in predation on onshore and ice‐associated prey types. We hypothesize that despite increasing use of onshore habitat by SB polar bears, lipid‐rich ice‐associated prey still remain the main foods sustaining this subpopulation. Nonetheless, we predicted that variability in feeding patterns has occurred over time in association with changing sea ice conditions.

## 2 METHODS

### 2.1 Study area and sampling

Capture and handling of polar bears in the SB subpopulation has occurred nearly every year from March to mid‐May over the past three decades (Atwood et al., 2016). The location of captures is on the sea ice off the north coast of mainland Alaska between Utqiaġvik and the border between Alaska and Yukon, Canada (157–141°W). In this region, subsistence harvesting of bowhead whales in the fall leaves behind remains (often referred to as “bone piles,” but that often consist of blubber, meat, viscera, as well as carcasses of other species; Herreman & Peacock, 2013) that provide a food source for polar bears coming ashore. These bone piles have been amassed at Point Barrow (until 2012), and Barter Island near the communities of Utqiaġvik and Kaktovik, respectively, as well as on Cross Island, approximately 20 km north of the Prudhoe Bay oil and gas field, and the location of the remains appears to influence the onshore distribution of SB polar bears (Atwood et al., 2016; Schliebe et al., 2008; Suydam & George, 2012; Wilson et al., 2017). Subcutaneous adipose tissue biopsies were collected from the rump of adult and subadult bears sampled in the spring of 2013–2016 (*n* = 125). Black guillemot specimens, used as a potentially representative seabird species for the region, were collected on Cooper Island (71°200N, 155°410W) in August of 2011, including eggs of various developmental stages (*n* = 12) and nestlings (*n* = 11).

Ancillary biological information for individual polar bears was collected at the time of sampling. Recorded information included position (lat/long) of capture, sex, straight‐line body length (straight from tip of muzzle to base of tail), and weight. Ages for first‐time independent captured bears were determined by counting the growth layer groups in the cementum of a vestigial premolar tooth (Calvert & Ramsay, 1998). Sex/age classes were designated as follows: adult females (AF) and males (AM; 5‐year‐olds and older), subadult females (SF) and males (SM; independent 2‐, 3‐, and 4‐year‐olds). Other age classes of polar bears were excluded due to the influence of nursing on their dietary tracer signatures (Polischuk, Hobson, & Ramsay, 2001).

### 2.2 Sea ice indices

Sea ice metrics for the entire SB region were calculated annually from daily ice statistics available from the National Snow and Ice Data Center (NSIDC; D. Douglas, unpublished data), as previously described (McKinney et al., 2014). Ice‐free days (IFD; measured as the period of time when ice concentration over the continental shelf first dropped below and last rose above 50% or 15%) and melt season (similarly defined as the length of time (days) between the first start of decline in ice concentration to the consistent increase in ice concentration over the continental shelf) were used as indicators of duration of reduced sea ice cover. Both IFD thresholds were used since 50% coverage is the most common threshold used for assessing polar bear habitat suitability, whereas 15% coverage is frequently used to define sea ice break‐up and freeze‐up dates (Atwood et al., 2016).

### 2.3 Fatty acid analysis

Fatty acid analysis was performed on polar bear adipose tissues, as well as homogenates of entire black guillemot eggs and nestlings. Adipose samples were stored at −80°C at the University of Connecticut, Storrs, CT, USA, prior to analysis. Oxidation has been shown to affect fatty acid signatures, particularly reducing the levels of long‐chained polyunsaturated fatty acids and increasing proportions of saturated and monounsaturated fatty acids (McKinney et al., 2017). Three samples visually appeared more yellow in color than others; however, their proportions of 22:6n3 were not significantly different than the samples that appeared white and fresh (*w* = 1,131.5, *p* = .87), thus it is unlikely that the fatty acid signatures of these bears were influenced by fatty acid oxidation. Lipids were extracted from the adipose and homogenate as previously described (McKinney et al., 2014, 2017). Fatty acids were then *trans*esterified to fatty acid methyl esters (FAME) and quantified as mass percentage of total FAME using gas chromatography with flame ionization detection.

### 2.4 Diet modeling

Diet estimates based on fatty acid signatures were determined via quantitative fatty acid signature analysis using the QFASA package (Iverson et al., 2018) in R (R Core Team, 2018). Estimates from QFASA were generated using calibration coefficients (CC) from mink (*Mustela vison*), a terrestrial carnivore model fed a marine diet, to adjust polar bear fatty acid proportions based on predictable changes relative to prey signatures due to predator biosynthesis and metabolism (Iverson, Field, Bowen, & Blanchard, 2004). Next, CC‐corrected polar bear signatures and prey signatures, including black guillemot (Table A1) as well as additional prey species used in previous SB studies, ringed seal (*n* = 89), bearded seal (*n* = 20), beluga whale (*n* = 29), and bowhead whale (*n* = 64; Budge, Springer, Iverson, Sheffield, & Rosa, 2008; Thiemann et al., 2008), were analyzed by QFASA using the Kullback‐Liebler distance measure. Previously published polar bear fatty acid signatures from 2004 to 2012 (McKinney et al., 2017) were also reanalyzed to include estimated proportions of black guillemot. Estimates were then combined with those from 2013–2016 to assess temporal trends in diet for this polar bear subpopulation. Implicit in this approach is the assumption that the prey fatty acid signatures have not substantially changed over the 2004–2016 time period; however, it was not feasible to test this by sampling all potential prey of SB polar bears in every year from 2004 to 2016. Thus, an important caveat to this study is that the modeled dietary variation based on the QFASA estimates may, at least in part, reflect other changes in the food web, for which we did not have a sufficient temporal span of prey collections to address.

Model diagnostics were performed for QFASA through simulation runs, as per Iverson et al. (2004). Prey‐on‐prey simulations were performed to evaluate how well prey species were distinguished from one another based on their fatty acid signatures. This model split the prey data randomly into “predator” and “prey” datasets. The “predator” was modeled using the “prey” to test if the model correctly identified the individual species relative to the others. Predator diet simulations were also performed to test how well the QFASA model output estimated a simulated diet. The simulation was run 100 times using the CCs and considering lipid composition (Iverson et al., 2004; Stewart & Field, 2011) to derive estimates from a “pseudo‐predator” whose fatty acid signature was generated from a given set of diet proportions closely reflecting the output of the QFASA diet estimates.

### 2.5 Statistical analysis

A permutation MANOVA (perMANOVA) using the vegan package for the R statistical environment (Oksanen et al., 2019) with a chi‐square distance‐based measure was used to assess the influence of sex/age class (i.e., adult male, adult female, subadult male, and subadult female) and capture year on the QFASA diet estimates, while considering the interaction year × sex/age class. The significant variables from the overall perMANOVA were then included in the perMANOVAs for the individual prey items by considering the diet proportions (*p_k_*, 1−*p_k_*), *k* = one of the five prey items. *p*‐Values were adjusted with the Holm method to account for multiple comparisons (Holm, 1979). For categorical variables that were significant based on the individual prey perMANOVA, pairwise perMANOVAs were used, with adjusted *p*‐values, to examine how variation in the proportions of individual prey items in polar bear diets differed among sex/age class. For continuous variables, Spearman's ranked correlations were performed. For the trend analysis, additional fatty acid signature results reported in McKinney et al. (2017) from 380 SB bears sampled in the spring 2004–2012 were reanalyzed to include the new prey species and then included. Separate perMANOVAs were also run that included one of the two sea ice indices, IFD or melt season, instead of capture year. The above statistical analyses were run for samples collected from 2013 to 2016 as well as the longer time series, 2004–2016. Statistical significance was set at *α* = 0.05 so results are significant if *p* ≤ .05.

## 3 RESULTS

### 3.1 Recent fatty acid‐based diet estimates

**Figure 1:**
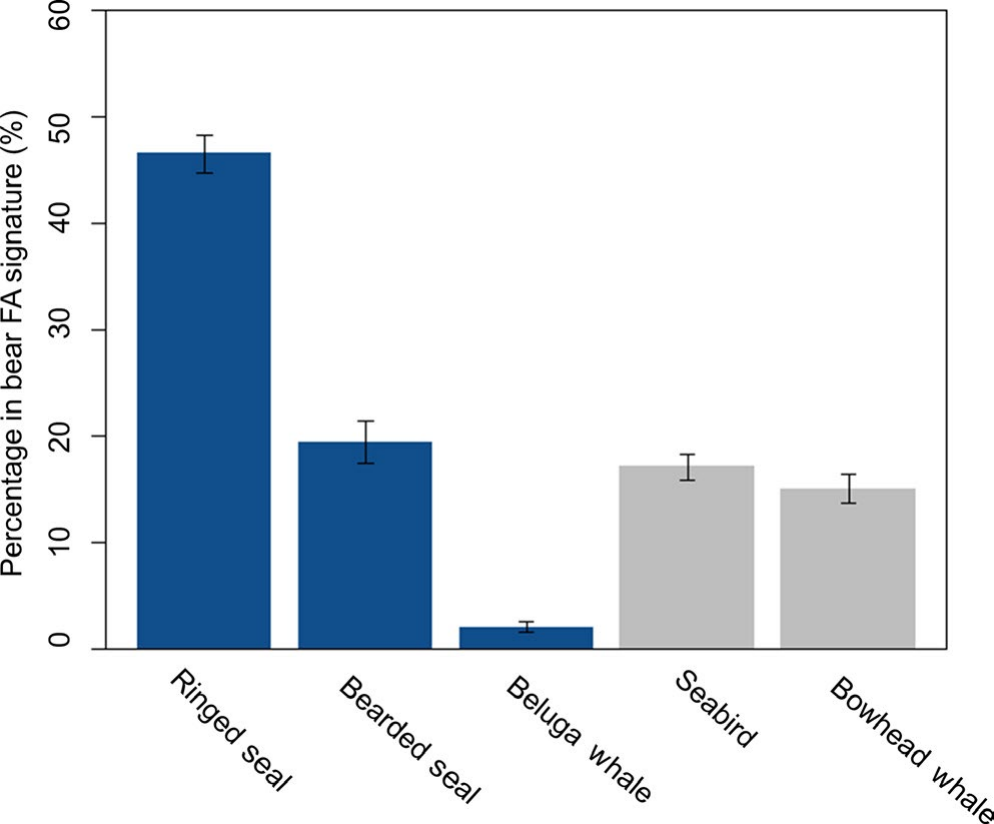
Mean (±*SE*) percentage of prey estimated by quantitative fatty acid signature analysis (QFASA) in the diets of southern Beaufort Sea (SB) polar bears from 2013 to 2016. Blue bars represent ice‐obligate prey and gray bars denote onshore food resources

QFASA‐based diet estimates from 2013 to 2016 suggested that ringed seal represented the largest proportion of polar bear diets (46.4 ± 1.8%). Estimates for other prey species were less than half of that estimated for ringed seal and were similarly lower among bearded seals (19.6 ± 2.0%), seabirds (17.0 ± 1.2%), and bowhead whales (15.0 ± 1.4%), and were lower again for beluga whales (2.0 ± 0.5%; Figure 1). A perMANOVA showed that variation in these diet estimates was significantly associated with capture year (*F*_3, 110_ = 2.75, *p* < .01) and with sex/age class (*F*_3, 101_ = 2.23, *p* = .02), but there was no interaction of year × sex/age class (*F*_8, 101_ = 0.86, *p* = .70).

### 3.2 Diet trends from 2004 to 2016 via fatty acids‐based diet estimates

**Figure 2:**
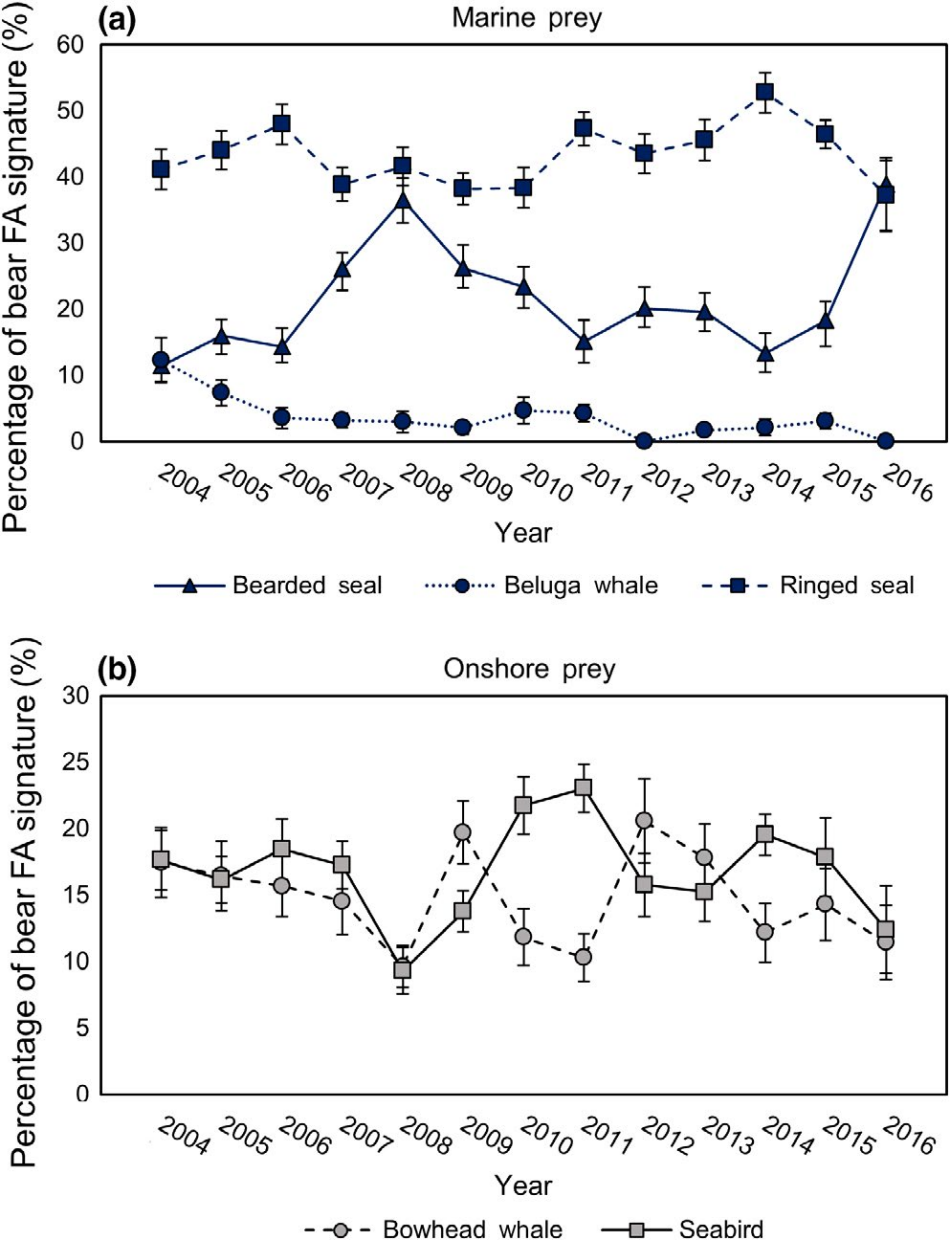
Relationship of mean (±*SE*) proportion of prey species estimated using quantitative fatty acid signature analysis (QFASA) in the diets of southern Beaufort Sea (SB) polar bears with capture year from 2004 to 2016 for (a) marine prey and (b) onshore prey resources demonstrating high interannual variability

**Figure 3:**
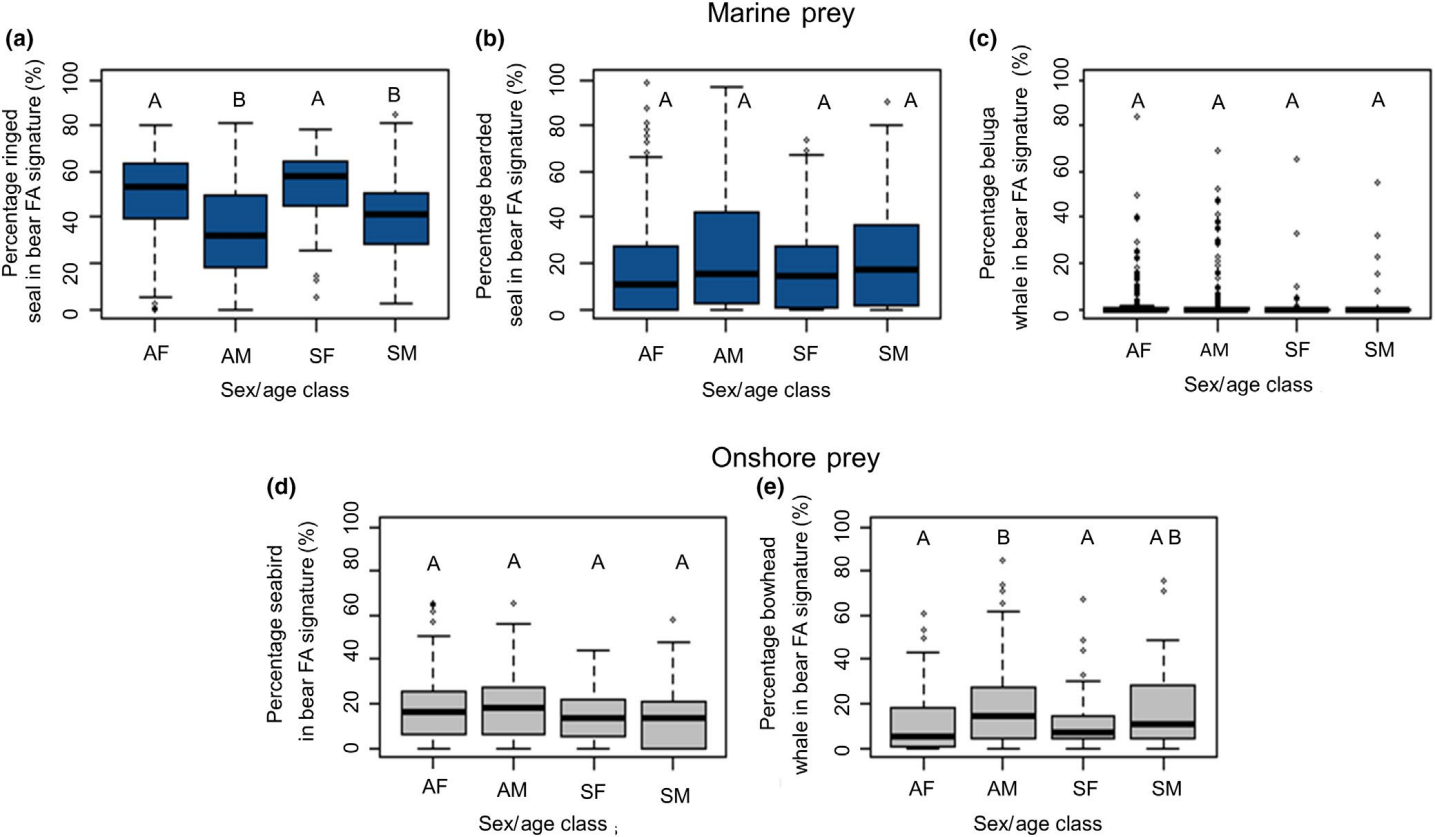
Percentage of individual prey species consumed as estimated using quantitative fatty acid signature analysis (QFASA) by sex/age class of southern Beaufort Sea polar bears sampled in 2004–2016, including ice‐associated or marine (prey in blue) (a) ringed seal, (b) bearded seal, and (c) beluga whale, and presumed onshore‐based (prey in gray) (d) seabirds and (e) bowhead whale carcass. Abbreviations used to denote sex/age class groups are as follows: AF = adult female, AM = adult male, SF = subadult female, SM = subadult male

The potential for decadal‐scale trends in SB polar bear diet estimates was determined using QFASA on polar bear samples collected from 2004 to 2016. An initial perMANOVA was run considering year, sex/age class, and the interaction of sex/age class with year. Diet varied with capture year (*F*_12, 456_ = 3.17, *p* < .001). The analysis additionally demonstrated significant differences in diet among sex/age classes (*F*_3, 456_ = 6.85, *p* < .001). There was significant and high interannual variation in estimated consumption of all prey species (*p* < .02; Figure 2); however, when annual means were analyzed for specific time trends only a (negative) relationship was found for beluga whale (*r*_s_ = −.76, *p* < .01), and no significant time trends were found for the other prey items (*p* > .30). Bowhead whale (*F*_3, 489_ = 6.39, *p* < .001), ringed seal (*F*_3, 489_ = 24.02, *p* < .001), and seabird (*F*_3, 489_ = 3.25, *p* = .04) estimates showed variation with sex/age class, while bearded seal and beluga whale estimates did not (*p* > .13). Adult male polar bears showed higher estimates for bowhead whale consumption than adult females and subadult females (*p* = .01). Conversely, adult males had lower estimates of ringed seal consumption than adult females and subadult females (*p* < .01). Subadult males also had lower levels of ringed seal estimated in their diets than adult females and subadult females (*p* < .01). Although significant sex/age class variation was found for the seabird estimates, no two demographic groups significantly differed (*p* > .06; Figure 3a–e).

For the time series from 2004 to 2016, subsequent perMANOVAs were performed in which year was replaced with one of the sea ice indices. There was a significant main effect of IFD at the 50% threshold and for melt season on polar bear diet estimates (*F*_1, 497_ = 3.85, *p* = .01, *F*_1, 497_ = 2.89, *p* = .02, respectively). The effect for IFD at 50% was shown for bearded seal estimates (*p* < .04), but IFD at the 50% threshold was not correlated with this prey item (*r*_s_ = 0.24, *p* = .43). The effect for melt season was not significant for any individual prey (*p* > .10). Significant main effects on SB polar bear diet estimates were not seen for the 15% threshold (*F*_1, 497_ = 2.23, *p* = .06). Interaction terms were not significant in models using the ice metrics (*p* > .54).

**Figure 4:**
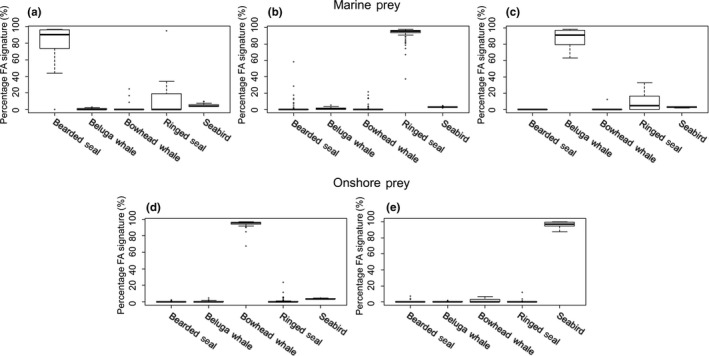
Boxplots of prey‐on‐prey simulation runs from quantitative fatty acid signature analysis (QFASA) using a prey library for southern Beaufort Sea polar bears showing results for (a) bearded seal, (b) ringed seal, (c) beluga whale, (d) bowhead whale, and (e) seabirds

The prey‐on‐prey simulation showed that the QFASA model was robust with respect to distinguishing between the prey species (Figure 4). The marine prey was mostly identified correctly, with correct identifications averaging 80.0 ± 24.0% for bearded seal (Figure 4a), 93.1 ± 8.1% for ringed seal (Figure 4b), and 88.1 ± 9.7% for beluga whale (Figure 4c). Although bearded seal identification accuracy was somewhat lower, it was mainly incorrectly identified as ringed seal, not as one of the onshore prey. Importantly, seabirds, which were not evaluated as prey in previous diet assessments for these polar bears, were well distinguished from the other prey species, averaging 96.5 ± 3.6% correct identifications in the simulation runs (Figure 4d). Bowhead whale also showed very high correct identifications in the simulations averaging 94.5 ± 4.1% of the runs (Figure 4e).

**Figure 5:**
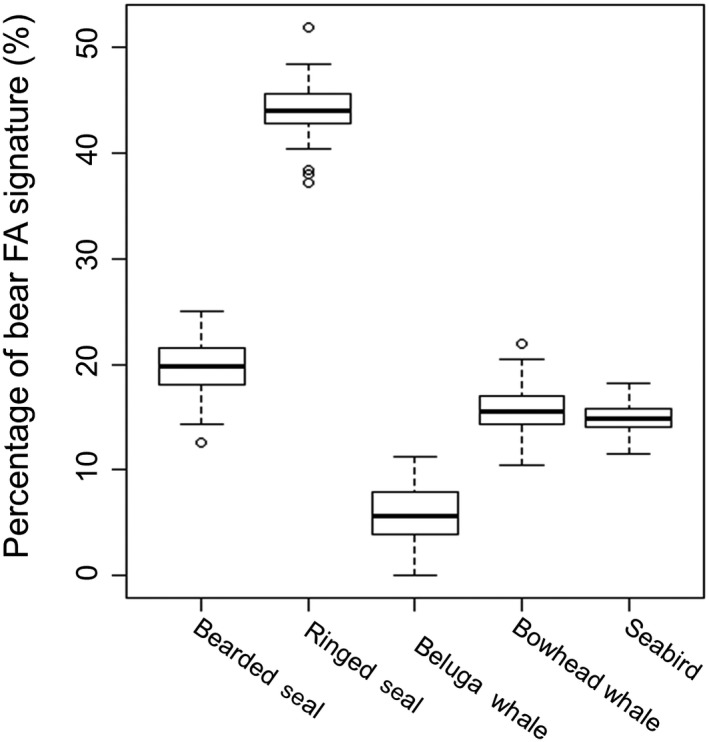
Results from pseudo‐predator simulations for quantitative fatty acid signature analysis (QFASA) of southern Beaufort Sea polar bears using calibration coefficients (CC) (Iverson et al., 2004). Boxplots show simulated data for the prey species given a “true” diet of 20% bearded seal, 45% ringed seal, 5% beluga whale, 15% bowhead whale, and 15% seabird

Predator simulations also supported the robustness of the QFASA model. We set the simulated “pseudo‐predator” diet to have a proportional composition of 20% bearded seal, 45% ringed seal, 5% beluga whale, 15% bowhead whale, and 15% seabird, proportions which were close to the those estimated by the model. The QFASA model performed well with the average estimates close to the simulated composition, specifically, the average estimates were 19.8 ± 2.1% for bearded seal, 44.8 ± 2.6% for ringed seal, 5.4 ± 2.6% for beluga whale, 15.0 ± 2.1% for bowhead whale, and 15.0 ± 1.3% for seabird (Figure 5).

## 4 DISCUSSION

Observations of predatory activities of individual polar bears in a small number of subpopulations have suggested substantial depredation of colonial nesting seabirds, particularly in recent years (Dey et al., 2017; Iverson et al., 2014; Smith et al., 2010, G. Divoky, unpublished data). However, the actual contribution of this onshore resource to the diets of polar bears at the subpopulation‐level has not been previously evaluated. Here, our estimates from QFASA suggested that seabirds comprised a moderate proportion of the biomass consumed by SB polar bears in recent years. The results are consistent with previous studies showing use of bowhead carcasses as a food resource (Herreman & Peacock, 2013; McKinney et al., 2017; Schliebe et al., 2008), and suggest that polar bears are also exploiting the substantial biomass of seabirds present off the Beaufort Sea coast (Drew, 2015). Importantly, SB polar bears, and adult females in particular, nonetheless still relied more heavily on their traditional major prey, ringed seal.

Although less than 1/5th of the diet, the seabird consumption estimate was somewhat higher than we anticipated, since observations of substantial depredation on seabird colonies have not been reported in this region, unlike elsewhere (Smith et al., 2010). It is possible that some of the model parameters, such as the prey lipid values, contributed to this outcome. Prey percent lipid values represent generalizations of what polar bears are consuming. For the seabird nestlings and eggs, we assumed that the prey is consumed in its entirety; therefore, percent lipid was input as that of the entire homogenate. For the marine mammals, 100% lipid was assumed as the contribution to diet, since polar bears prefer high‐energy blubber relative to muscle or other tissues; nonetheless, this simplifying assumption could represent an overestimate of the amount of lipid actually consumed from these prey species. Thus, we reran the model at 80% lipid for marine mammals (as polar bears may consume an ~80:20 lipid:protein diet; Cherry et al., 2011) to test the sensitivity of the model to this parameter. We found that the diet estimates (43.6 ± 1.0% ringed seal, 21.8 ± 1.2% bearded seal, 14.8 ± 0.6% seabird, and 15.2 ± 0.8% bowhead and 4.6 ± 0.6% beluga) were very similar to the original estimates, indicating that the model results were not sensitive to the lipid parameter. Another potential caveat in terms of our modeling results is that the marine mammal fatty acid data were from samples collected in earlier years, and if food web changes have occurred (Divoky et al., 2015; Harwood et al., 2015), previous fatty acid signatures may not be fully representative of current prey fatty acid signatures. To assess this possibility, temporal data for all potential prey species would be needed; unfortunately, such a large‐temporal sampling of multiple potential prey was outside the scope of this study. Still, the prey‐on prey simulation indicated that the QFASA model performed well at distinguishing seabird fatty acid signatures from those of other prey species; thus, the seabird estimates were, at least, not biased by similarity of their signatures to other prey. Regardless, the quantitative estimate of seabirds in the diet of SB polar bears should be interpreted with caution, given the aforementioned assumptions and uncertainties in the model parameters.

Black guillemot are only one of the many subsurface, pelagic‐feeding Arctic seabirds that may be susceptible to polar bear predation, although just the presence of the birds does not mean they are vulnerable to predation (Kuletz et al., 2015). Breeding colonies may be important in terms of foraging efficiency. Although polar bears have been observed catching birds in the sea (Stempniewicz, Kidawa, Barcikowski, & Iliszko, 2014), they have mainly been seen foraging upon eggs and nestlings and are seen most often eating large quantities at once (Divoky et al., 2015; Iverson et al., 2014). Although not a seabird, flightless molting adult lesser snow geese (*Chen caerulescens caerulescens*) have been hunted in WHB and other subpopulations (Iles et al., 2013). The phenology of these foraging strategies requires that the breeding or molt seasons of the birds coincide with when polar bears come ashore in response to sea ice conditions (Iverson et al., 2014). Besides the Cooper Island colony, there are other seabird breeding sites in the SB region, including black guillemot on Herschel Island and thick‐billed murre colonies on the cliff‐like habitat near Cape Parry (Dickson & Gilchrist, 2002), though these colonies do not have the documented predation seen on Cooper Island (Divoky et al., 2015). The SB region is also a key staging area for hundreds of thousands of ducks in the spring, as well as a molting area for ducks, mergansers, scaup, and scoters by the tens of thousands in the summer (Dickson & Gilchrist, 2002). Furthermore, seabirds such as common eiders are also known to breed in the area and many species breed in the coastal plain of the Arctic National Wildlife Refuge (Goudie, Robertson, & Reed, 2000; U.S. Fish & Wildlife Service, 2015). We used available eggs and nestlings from black guillemot to represent a proxy for other pelagic‐feeding seabirds who may have similar fatty acid signatures. Although in terms of seabirds in the northern Alaskan region, only fatty acid signatures for black guillemot have previously been published (Budge et al., 2008), direct diet knowledge suggests that the diets of black guillemot may overlap with other pelagic seabirds in the region. That is, according to the North Pacific Seabird Data Portal (World Seabird Union, 2019), in addition to black guillemot, other seabirds in the region include (but are not necessarily limited to) common eider, Steller's eider (*Polysticta stelleri*), spectacled eider (*Somateria fischeri*), glaucous gull (*Larus hyperboreus*), Arctic tern (*Sterna paradisaea*), and Sabine's gull (*Xema sabini*). Black guillemot consume small fish (mainly nearshore, benthic) and crustaceans, while Arctic tern consume mainly small fish and crustaceans, eider eat mollusks and some small fish and crustaceans, Sabine's gull eat small fish and crustaceans and insects, and glaucous gull eat many foods, including small fish, crustaceans and mollusks (National Audubon Society, 2019). Thus, there is similarity in the diets among these seabird species, which lends support to our assumption that black guillemot fatty acid signatures may be reasonably representative of some of the other seabird prey in the region, at least in this first attempt to quantify seabirds in the diet of polar bears. Nonetheless, other potentially important seabirds and geese should be included in future modeling; inclusion of such potential prey could identify seabird populations potentially at risk due to polar bear predation.

A question that arises from our estimate of seabird proportions in the SB polar bear diet, is how much this estimate might represent relative to the seabird biomass in the region? Pagano et al. (2018) recently estimated a 12,000 kcal/day requirement for polar bears, which is equivalent to 1.3 kg fat or 3.0 kg of protein per day, assuming 1 g of fat supplies 9 kcal and 1 g of protein supplies 4 kcal. At a diet of 80:20 fat:protein (Cherry et al., 2011), though, this is 1.0 kg of fat and 0.6 kg of protein per polar bear per day. Focussing on the 1.0 kg fat requirement for simplicity, for 900 polar bears, this would be 900 kg of fat per day, or 328 thousand kg fat per year. At ~17% of the subpopulation's diet estimated for seabirds (our study), this would represent 55 thousand kg fat per year. As seabirds and their eggs and nestlings are only part fat, say 10% or so (we found 0.2%–20% lipid in the black guillemot nestlings and eggs), this means that the bears would be consuming 550 thousand kg of seabirds per year. This represents perhaps about 4% of the seabird biomass in the 50,000 km^2^ region (based on our ~14 million kg estimate), and thus likely a very small portion of the biomass of seabirds in the entire SB polar bear habitat. Prop et al. (2015) reported that waterfowl and seabird nest predation in Svalbard and east Greenland exceeded 90% in some years, which suggests bears are able to efficiently exploit this seasonal resource when it becomes available.

Temporal trends analysis did not show clear increases or decreases in proportional consumption of any prey species other than beluga, which were uncommon in the diet, despite significant interannual variation for all prey in the diet. The continued high interannual variability, similar to that reported in the earlier study (McKinney et al., 2017), suggests the critical importance of continued long‐term annual monitoring to provide sufficient power to detect ecological change within such a highly variable system. Our study also showed IFD and melt season affect polar bear diet, supporting our hypothesis that sea ice loss is having a discernable effect on polar bear feeding ecology.

Males showed the most varied diet. This finding may be related to their foraging more often near open water on ice floes (Stirling, Andriashek, & Calvert, 1993). As well, males are larger, making it possible to successfully hunt larger prey in addition to ringed seal (Stirling & Archibald, 1977). Males consumed, on average, lower proportions of ringed seal than adult and subadult females, consistent with males making use of other prey items including onshore prey. These results agree with the finding that adult males make up the highest proportion of fall onshore surveys (Atwood et al., 2015). Additionally, consumption of bowhead whale was previously shown to be positively associated with body condition, at least for males (McKinney et al., 2017). This implies that males, in particular, are more successful in using the bowhead carcasses, and therefore may benefit more from lower energy expended in using this food resource.

As the proportion of SB polar bears coming ashore and using nearshore/onshore habitat increases with sea ice retreat, it is crucial to assess if those sampled on sea ice and within the study area in spring remain a representative subsample of the entire subpopulation. It is possible that bears that spend summer and fall on land also display fidelity to the nearshore region at other times of year, as satellite telemetry data indicated SB polar bears that fed on bowhead whale had spent 90% of the previous year within 50 km of the coastline (e.g., Rogers et al., 2015). This disproportionate use of nearshore sea ice habitats may increase their probability of capture in spring and lead to population‐level estimates of the use of onshore food items that are biased high. Yet, onshore behavior in SB polar bears has been linked to social learning or genetic inheritance, indicating that future generations of bears using the onshore habitat will continue to use these resources as sea ice loss progresses (Lillie, Gese, Atwood, & Sonsthagen, 2018). If onshore habitat use results in improved fitness (reproductive output or survival) as seen with body condition, the behavior will also likely proliferate.

This study represents the first attempt to quantify seabird as prey in the diet of polar bears and has only one potentially representative avian species; therefore, we recommend further research to quantify the different seabird species and other potential onshore prey susceptible to polar bear predation and at what rates, as these pieces of evidence would suggest how onshore foraging by polar bears may impact seabird populations. Although our study has suggested that they may only represent a relatively minor component of polar bear diets, devastation of breeding colonies by polar bear predation has been reported (Dey et al., 2017; Iverson et al., 2014; Smith et al., 2010), and if sustained over multiple breeding seasons, creates the potential for localized reproductive failure.

## CONFLICT OF INTEREST

None declared.

## AUTHORS' CONTRIBUTIONS

TCA and MAM designed the work. JB, TCA, and GJD acquired the data. JB and CS analyzed the data. JB, TCA, GJB, CS, and MAM interpreted the data. JB drafted the work, and TCA, GJD, CS, and MAM critically revised the work.

## Data Availability

Fatty acid signature and diet estimate data are publicly archived at https://alaska.usgs.gov/portal/.

## Appendix 1

**Table A1:** Mean (±*SE*) fatty acid profiles (mass % of total) for homogenate black guillemot (*Cepphus grylle mandtii*) eggs and nestlings from Cooper Island

Fatty acid (“dietary”)	Mean ± *SE*	Fatty acid (“dietary”)	Mean ± *SE*
*Saturated fatty acids (SFA)*		*Polyunsaturated fatty acids (PUFA)*	
12:0	0.03 ± 0.01	**16:2n‐6**	**0.02 ± 0.00**
13:0	0.00 ± 0.00	**16:2n‐4**	**0.10 ± 0.01**
14:0	0.39 ± 0.04	**16:3n‐6**	**0.06 ± 0.01**
iso15:0	0.02 ± 0.00	**16:3n‐4**	**0.06 ± 0.05**
anti15:0	0.01 ± 0.00	**16:4n‐3**	**0.06 ± 0.00**
15:0	0.09 ± 0.01	**16:4n‐1**	**0.68 ± 0.13**
iso16:0	0.03 ± 0.01	18:2Δ5,11	0.04 ± 0.00
16:0	17.52 ± 0.68	18:2n‐7	0.02 ± 0.00
7Me16:0	0.14 ± 0.01	**18:2n‐6**	**0.93 ± 0.10**
iso17:0	0.05 ± 0.00	**18:2n‐4**	**0.08 ± 0.01**
17:0	0.18 ± 0.01	**18:3n‐6**	**0.04 ± 0.01**
18:0	10.69 ± 0.92	**18:3n‐4**	**0.05 ± 0.01**
20:0	0.50 ± 0.08	**18:3n‐3**	**0.15 ± 0.01**
*Monounsaturated fatty acids (MUFA)*		**18:3n‐1**	**0.03 ± 0.00**
14:1n‐9	0.03 ± 0.00	**18:4n‐3**	**0.09 ± 0.02**
14:1n‐7	0.01 ± 0.00	**18:4n‐1**	**0.09 ± 0.01**
14:1n‐5	0.02 ± 0.00	**20:2n‐6**	**0.55 ± 0.13**
15:1n‐8	0.00 ± 0.00	**20:3n‐6**	**0.32 ± 0.09**
15:1n‐6	0.00 ± 0.00	**20:4n‐6**	**5.42 ± 0.81**
16:1n‐11	0.15 ± 0.01	**20:3n‐3**	**0.48 ± 0.18**
16:1n‐9	0.52 ± 0.04	**20:4n‐3**	**0.15 ± 0.07**
16:1n‐7	3.25 ± 0.42	**20:5n‐3**	**3.96 ± 0.51**
16:1n‐5	0.03 ± 0.00	**21:5n‐3**	**0.03 ± 0.01**
17:1	0.13 ± 0.01	**22:4n‐6**	**0.41 ± 0.08**
18:1n‐11	0.23 ± 0.02	**22:5n‐6**	**0.09 ± 0.02**
18:1n‐9	34.9530 ± 2.55	**22:4n‐3**	**0.01 ± 0.00**
18:1n‐7	5.58 ± 1.02	22:5n‐3	1.23 ± 0.09
18:1n‐5	0.43 ± 0.03	**22:6n‐3**	**5.81 ± 0.41**
**20:1n‐1**	**20.45 ± 0.06**		
**20:1n‐9**	**1.67 ± 0.15**	∑SFA	29.6 ± 0.58
**20:1n‐7**	**0.40 ± 0.05**	∑MUFA	49.2 ± 2.53
**22:1n‐11**	**0.17 ± 0.04**	∑PUFA	21.02 ± 2.29
**22:1n‐9**	**0.14 ± 0.02**	∑n‐3 (omega‐3)	12.0 ± 1.12
**22:1n‐7**	**0.04 ± 0.01**	∑n‐6 (omega‐6)	7.88 ± 1.16
